# Mindfulness and academic alienation among university students: the mediating roles of cognitive fusion and psychological resilience

**DOI:** 10.3389/fpsyg.2026.1813062

**Published:** 2026-04-10

**Authors:** Bingying Duan, Shuting Liao, Huilin Wang

**Affiliations:** 1 School of Business, Hunan University of Science and Technology, Xiangtan, China; 2 Hunan Industry Polytechnic, Changsha, China; 3 International College, National Institute of Development Administration, Bangkok, Thailand

**Keywords:** academic alienation, cognitive fusion, mindfulness, psychological resilience, university students

## Abstract

**Introduction:**

Academic alienation has become an increasingly salient issue among university students, as it is closely related to students’ engagement with learning and their psychological adjustment in academic contexts. Mindfulness has been widely discussed as a psychological characteristic associated with individuals’ awareness and regulation of internal experiences, yet the processes linking mindfulness to academic alienation remain insufficiently examined. In particular, limited attention has been given to the potential roles of cognitive fusion and psychological resilience in shaping students’ academic experiences.

**Methods:**

A paper-based survey was administered to 569 undergraduate students from three universities in Hunan Province, China. Measures included mindfulness, cognitive fusion, psychological resilience, and academic alienation. Structural equation modeling (SEM) was conducted using IBM SPSS AMOS 26, with confirmatory factor analysis (CFA) evaluating the measurement model. Indirect effects were tested via bootstrapping.

**Results:**

Mindfulness showed a negative association with cognitive fusion and a positive association with psychological resilience. Cognitive fusion was positively associated with academic alienation and negatively associated with psychological resilience, whereas psychological resilience was negatively associated with academic alienation. Further analyses indicated that cognitive fusion and psychological resilience mediated the association between mindfulness and academic alienation.

**Conclusion:**

The findings offer insight into the psychological processes associated with university students’ experiences in academic settings. By highlighting the relevance of cognitive and resilience-related factors in relation to academic alienation, the results help clarify how students’ internal psychological functioning may be linked to their engagement with learning. These observations may inform future research examining students’ academic adjustment and well-being.

## 1 Introduction

The university stage is a critical period for the development of individuals’ professional competence, social role identity, and social adaptability (Briggs et al., 2012). During this period, students’ physical and psychological well-being, together with their learning experiences, are closely associated with the achievement of the Sustainable Development Goals (SDGs), particularly SDG 3 (Good Health and Well-Being) and SDG 4 (Quality Education) (United Nations, 2015). However, as academic demands intensify and learning environments become increasingly complex, some university students gradually experience a sense of psychological detachment from school and academic activities, commonly referred to as academic alienation (Hascher and Hagenauer, 2010). Alienation is generally regarded as a psychological state that arises when individuals find it difficult to adapt to social or situational demands, and it may trigger a series of negative emotional and behavioural responses (Kumari and Kumar, 2017). More specifically, academic alienation encompasses a cluster of negative learning-related attitudes, such as helplessness, meaninglessness, normlessness, and social isolation (Brown et al., 2003). These psychological states not only diminish students’ engagement in learning but may also contribute to later difficulties in academic adjustment.

Students with a tendency towards academic alienation often display a series of negative characteristics at both behavioural and psychological levels. Specifically, such students may gradually lose interest in learning, lack motivation, and show reduced participation in classroom and school activities, such as absenteeism, low classroom participation, or perfunctory completion of assignments (Morinaj et al., 2023; Morinaj et al., 2017). At the same time, they tend to interact less with teachers and classmates, struggle to establish stable and effective interpersonal relationships, and find it difficult to develop a sense of belonging (Karatepe et al., 2021). In addition, students experiencing academic alienation often question the value and meaning of university learning, believing that what they learn is of limited relevance to their future development (Mau, 1989). Academic alienation has therefore become an important issue in higher education because it not only hinders academic development, but is also associated with lower subjective well-being (Morinaj and Hascher, 2019), poorer academic performance (Yirci and Kalaylı, 2024), and higher academic anxiety (Khangura et al., 2020b). These findings suggest that academic alienation is not merely a temporary negative attitude, but a maladaptive academic state with consequences for both students’ functioning and emotional adjustment (Morinaj and Hascher, 2019; Yirci and Kalaylı, 2024; Khangura et al., 2020b). Therefore, identifying modifiable psychological factors associated with academic alienation is both theoretically and practically important.

Existing studies suggest that the factors associated with academic alienation can mainly be grouped into social and psychological domains. At the social level, previous studies have highlighted contextual and relational influences, such as teaching style and classroom composition (Hadjar and Backes, 2023), school culture (Ghalavandi, 2023), academic capitalism (Poutanen, 2023), teachers’ servant leadership (Atiq et al., 2025), disliked majors, lack of social spaces, and teachers’ positions on campus (Atila, 2023). At the psychological level, relevant factors include low academic self-efficacy (Hascher and Hagenauer, 2010), life satisfaction (Chang et al., 2025), and self-dissociation (Adam, 2023). Despite this growing body of research, the association between mindfulness and academic alienation remains insufficiently examined. In particular, how mindfulness may shape students’ experience of alienation through specific psychological mechanisms has not yet been systematically clarified.

Mindfulness, originally rooted in Buddhist contemplative traditions and later developed within Western psychology, is commonly conceptualised as a present-centred and non-judgemental awareness of ongoing internal and external experiences (Kabat-Zinn, 2003; Bishop et al., 2004). Existing research has shown that mindfulness-based interventions are associated with beneficial outcomes among university students, particularly in relation to mental health and well-being (Chiodelli et al., 2022). Focusing on the present helps students concentrate their attention on current academic tasks and reduce interference from irrelevant thoughts or emotions, which may facilitate learning engagement (Bishop et al., 2004). Enhancing self-awareness helps students perceive and identify their own emotions, thoughts, and feelings, and accept them without judgment, thereby avoiding unnecessary psychological distress (Bishop et al., 2004; Keng et al., 2011). In the present study, mindfulness is not treated as the simple opposite of “living in the past.” Rather, it is conceptualised as a present-centred attentional and emotional-regulatory capacity (Bishop et al., 2004; Chambers et al., 2009). The argument of this study is that when students have lower present-centred awareness, they may be more vulnerable to becoming entangled with negative academic thoughts and emotions, which may subsequently undermine adaptation to academic demands and increase the likelihood of academic alienation (Krafft et al., 2019; Dong et al., 2021; García-Gómez et al., 2019). For this reason, mindfulness is introduced here as a potential antecedent psychological resource rather than as a direct temporal explanation of alienation.

One possible mechanism underlying this association is cognitive fusion. Cognitive fusion is a central construct within Acceptance and Commitment Therapy and describes a pattern in which individuals become overly entangled with their thoughts, allowing verbal processes to rigidly guide behavior (Hayes et al., 2006). In this state, thoughts are experienced as literal truths rather than temporary mental events (Bardeen and Fergus, 2016). When a person interprets a self-critical thought as an objective reality, it may trigger avoidance responses and exaggerated reactions (Hayes et al., 1999). Empirical research has linked cognitive fusion to heightened psychological distress, including symptoms of anxiety and depression (Chen et al., 2023; Bardeen and Fergus, 2016), as well as to reduced psychological flexibility (Hayes et al., 1996; Hayes et al., 1999). University students appear particularly vulnerable to fusion-related patterns (Miniati et al., 2023), and elevated levels of fusion have been associated with depressive symptoms, generalized and social anxiety, hostility, academic distress, and problems related to the student role (Krafft et al., 2019). In contrast, mindfulness encourages an open and accepting awareness of present-moment experiences, allowing individuals to observe thoughts without becoming entangled in their content (Bishop et al., 2004). By fostering self-awareness and psychological distance from internal experiences, mindfulness may reduce cognitive rigidity and strengthen psychological flexibility (Hayes et al., 2006). Consistent with this mechanism, participation in mindfulness training has been linked to lower levels of cognitive fusion (Tanay et al., 2012). In the present study, cognitive fusion refers more specifically to students’ tendency to over-identify with negative academic thoughts, such as self-doubt, fear of failure, and negative interpretations of learning experiences, and to treat these thoughts as objective facts (Krafft et al., 2019; Hayes et al., 2006).

Another possible mechanism is psychological resilience. Although psychological resilience has been conceptualized in multiple ways, most accounts converge on two essential elements: exposure to adversity and successful adaptation (Luthar et al., 2000). In other words, resilience implies the capacity to maintain or regain psychological functioning when facing stress and challenge (Vella and Pai, 2019). As higher education expands and social competition intensifies, university students are increasingly exposed to sustained academic demands arising from both internal expectations and external environmental pressures (Ramón-Arbués et al., 2020). Prolonged exposure to such pressures may contribute to withdrawal tendencies, reduced investment in academic tasks, and emotional as well as physical exhaustion, gradually diminishing students’ enthusiasm for learning (Kent et al., 2022). Previous studies indicate that mindfulness is positively associated with resilience (Zhang et al., 2023; Pidgeon and Keye, 2014). Through mindfulness practice, individuals learn to observe intrusive thoughts and emotions as transient experiences rather than enduring realities (Shapiro et al., 2006), which enhances tolerance of unpleasant internal states and strengthens their capacity to cope with stressful or adverse circumstances (Leontopoulou, 2006). In the present study, psychological resilience refers to students’ adaptive capacity to deal successfully with academic setbacks and challenges, regulate stress, and remain constructively engaged with learning demands (Martin and Marsh, 2008; Romano et al., 2021). Related literature further suggests that self-compassion may serve as a relevant emotional-regulatory resource in this process, because a less self-critical and more accepting stance towards personal difficulties is associated with more adaptive coping and greater resilience, and may also be relevant to how individuals relate to distressing thoughts rather than becoming over-identified with them (Neff, 2003; Bluth et al., 2018; Allen and Leary, 2010; Böge et al., 2022). Although self-compassion is not included as a focal variable in the present model, acknowledging this perspective helps situate the current framework within the broader literature on adaptive emotion regulation.

At the same time, cognitive fusion may weaken psychological resilience. Psychological resilience stems from a cognitive processing regulatory system that can flexibly adjust attentional bias, executive control, and cognitive interpretation styles according to the situation, thereby promoting adaptive recovery in adversity (Parsons et al., 2016). Individuals with cognitive fusion are excessively troubled by negative thoughts and cannot flexibly regulate their behavior based on real situational feedback, thereby restricting adaptive responses (Hayes et al., 2006). In a state of cognitive fusion, individuals may exhibit more rigid patterns of responding to internal experiences, which appears inconsistent with the cognitive flexibility that supports resilient adaptation (Hayes et al., 2006; Yao and Hsieh, 2019). Accordingly, students who are highly fused with negative academic thoughts may find it more difficult to respond adaptively to setbacks, reinterpret challenges constructively, and sustain engagement in valued academic activities (Juszczyk-Kalina et al., 2025; Palladino et al., 2013; Watanabe and Akechi, 2023).

Cognitive fusion and psychological resilience may, in turn, be closely related to academic alienation. Academic alienation is generally understood as a maladaptive learning-related state shaped by cognitive and emotional processes (Hascher and Hadjar, 2018). When students perceive learning as meaningless, lacking control, or unrelated to their personal development, they tend to gradually distance themselves psychologically from learning activities (Hascher and Hagenauer, 2010), and over time develop a general negative tendency toward school learning (Hascher and Hadjar, 2018). Cognitive fusion emphasizes individuals’ high degree of fusion with internal experiences. When students are highly fused with negative learning-related thoughts, these thoughts are more likely to be regarded as objective facts (Hayes et al., 1999), thereby solidifying into negative views toward learning. At the same time, cognitive fusion also makes students more likely to avoid negative emotions related to learning, thereby possibly escaping learning tasks (Hayes et al., 2006). In contrast, psychological resilience represents students’ adaptive capacity to manage academic setbacks and sustained learning pressures (Martin and Marsh, 2006). Evidence suggests that higher resilience is associated with lower levels of academic alienation (Khangura et al., 2020a). Resilient students tend to demonstrate stronger academic self-efficacy (Beri and Kumar, 2018), greater engagement in learning activities, and more positive academic adjustment (Cassidy, 2015). Through these mechanisms, resilience may buffer against the development of alienation. In this study, academic alienation is therefore treated not simply as a general negative feeling, but as a relatively stable state of disengagement from academic tasks, relationships, and meanings, shaped by how students interpret and regulate their academic experiences (Hascher and Hadjar, 2018; Vafa et al., 2021).

Taken together, mindfulness may be associated with academic alienation not only directly, but also indirectly through cognitive fusion and psychological resilience. Prior research has frequently examined cognitive fusion and psychological resilience in relation to negative emotional outcomes such as anxiety and depression (Cookson et al., 2020; Liu et al., 2021). In academic contexts characterized by sustained pressure, students may become increasingly entangled with evaluative or self-critical thoughts, resulting in heightened cognitive fusion (Miniati et al., 2023). Such rigid cognitive patterns constrain flexible behavioral regulation and weaken adaptive coping capacity, thereby diminishing psychological resilience (Parsons et al., 2016). Reduced resilience, in turn, may foster feelings of helplessness and disengagement from learning, ultimately contributing to academic alienation (Karatepe et al., 2021). By cultivating present-centered awareness and non-reactive observation, mindfulness enables individuals to relate to internal experiences without excessive identification (Bishop et al., 2004; Hölzel et al., 2011). This decentering process reduces fusion with negative thoughts (Tanay et al., 2012) while simultaneously strengthening psychological resilience (Zhang et al., 2023; Pidgeon and Keye, 2014). The logic of the present model is therefore sequential rather than merely descriptive: mindfulness is proposed to operate as an antecedent attentional-regulatory resource; lower cognitive fusion is expected to reduce students’ domination by negative academic thoughts; and this may help preserve or strengthen psychological resilience, which in turn reduces the likelihood of academic alienation. In this way, the mediating pathways proposed in the present study are grounded in a theoretically connected process rather than in a set of isolated pairwise associations.

In view of the deficiencies in the above research field, this study aims to: (1) explore the psychological mechanism of academic alienation among university students; (2) examine the relationships among mindfulness, cognitive fusion, psychological resilience, and academic alienation; and (3) propose targeted suggestions to reduce university students’ tendency toward academic alienation and promote their physical and mental health development.

Based on the above literature review and theoretical reasoning, the following hypotheses are proposed:

*Hypothesis 1 (H1)*: Mindfulness is negatively associated with cognitive fusion.

*Hypothesis 2 (H2)*: Mindfulness is positively associated with psychological resilience.

*Hypothesis 3 (H3)*: Cognitive fusion is negatively associated with psychological resilience.

*Hypothesis 4 (H4)*: Cognitive fusion is positively associated with academic alienation.

*Hypothesis 5 (H5)*: Psychological resilience is negatively associated with academic alienation.

*Hypothesis 6 (H6)*: Cognitive fusion and psychological resilience mediate the relationship between mindfulness and academic alienation.

**Figure 1:**
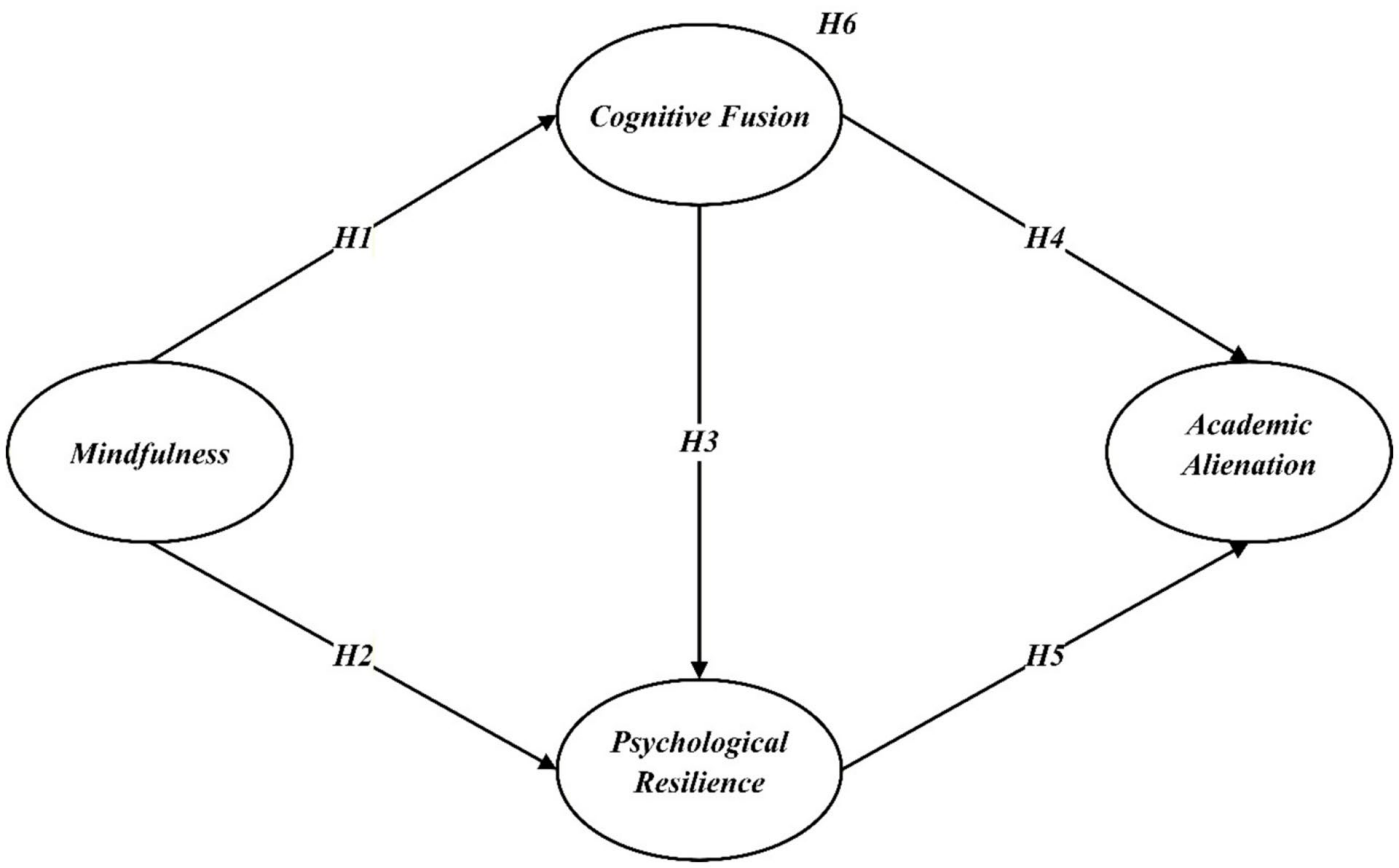
Hypothesized research model.

A summary of all hypotheses is presented in Figure 1.

## 2 Methodology

### 2.1 Participants and pro**ced**ure

The sample comprised undergraduate students from three universities located in Hunan Province, China. Data were collected in December 2025 through a paper-and-pencil survey using a convenience sampling strategy. The inclusion criteria were as follows: participants had to be currently enrolled full-time undergraduate students at one of the three participating universities, able to understand the questionnaire content, and willing to participate voluntarily in the study. The exclusion criteria included non-undergraduate respondents, questionnaires returned without consent, and questionnaires with substantial missing data or clearly invalid response patterns (e.g., uniform responses across all items). A total of 600 questionnaires were distributed (200 per university). After screening for incomplete or invalid responses, 569 usable questionnaires remained, resulting in a response rate of 94.8%.

Participation was voluntary. Prior to completing the survey, students were informed of the study’s academic purpose and assured of anonymity and confidentiality; no identifying information was recorded. Surveys were administered in classroom settings under the supervision of trained research assistants to maintain standardized procedures and data quality.

Demographic information is summarized in Table 1. Among the 569 participants, 56.1% were female and 43.9% were male. First-year students represented the largest group (37.6%), followed by fourth-year (23.9%), third-year (19.5%), and second-year students (19.0%), indicating a relatively balanced distribution across academic years. In terms of academic majors, 45.7% were from Humanities and Social Sciences, 42.4% from Science and Engineering, and 12.0% from Arts and Physical Education. Regarding perceived family economic status, most students reported an average level (65.2%), while smaller proportions indicated relatively difficult (18.3%), very difficult (3.7%), relatively affluent (12.1%), or very affluent (0.7%) backgrounds. Additionally, 22.7% of participants reported holding student leadership positions.

**Table 1:** Demographic characteristics (*N* = 569).

Variable	Category	*n*	%
Gender	Male	250	43.9
	Female	319	56.1
Grade level	First year	214	37.6
	Second year	108	19.0
	Third year	111	19.5
	Fourth year	136	23.9
Field of study	Humanities and Social Sciences	260	45.7
	Science and Engineering	241	42.4
	Arts and Physical Education	68	12.0
Family economic status	Very difficult	21	3.7
	Relatively difficult	104	18.3
	Average	371	65.2
	Relatively affluent	69	12.1
	Very affluent	4	0.7
Student leadership role	Yes	129	22.7
	No	440	77.3

### 2.2 Instruments

The survey consisted of five sections. The first section gathered demographic information, including gender, academic year, major, family economic status, and student leadership experience.

Mindfulness was assessed with five items adapted from the Mindful Attention Awareness Scale (MAAS) (Van Dam et al., 2010). The MAAS was originally developed as a unidimensional measure of present-moment attention and awareness, and its Chinese version has shown satisfactory reliability and validity in college populations (Deng et al., 2012). In the present study, we used the 5-item version because Van Dam et al. (2010) showed, through item response theory analyses, that these items provided the strongest discrimination across levels of trait mindfulness. In addition, because the questionnaire was administered in classroom settings and included multiple scales, the brief version helped reduce respondent burden while preserving the core attentional-awareness content of the original MAAS. The selected items are behavior-based and linguistically straightforward, which makes them suitable for Chinese university students in paper-based survey research. An example item is: “I am able to maintain clear awareness of what I am doing.”

Cognitive fusion was measured using the Cognitive Fusion Questionnaire (CFQ) (Gillanders et al., 2014), which includes seven items evaluating the extent to which individuals become entangled with their thoughts and have difficulty distancing themselves from internal experiences (e.g., “I tend to get very entangled in my thoughts”).

Psychological resilience was assessed with the Brief Resilience Scale (BRS) (Smith et al., 2008), comprising six items that reflect individuals’ ability to recover from stress or adversity (e.g., “I tend to bounce back quickly after hard times”).

Academic alienation was measured using the alienation-from-learning subscale of the School Alienation Scale (SALS) (Morinaj et al., 2020). This choice was theoretically intentional. School alienation has been conceptualized as a multidimensional construct comprising alienation from learning, teachers, and classmates, and these domains are related but distinct rather than interchangeable indicators of a single higher-order factor (Hascher and Hadjar, 2018; Morinaj et al., 2020). Because the present study focuses specifically on academic alienation, that is, students’ psychological detachment from learning tasks, academic meaning, and engagement with study, the learning dimension provided the closest conceptual match to the focal outcome variable. By contrast, the classmates and teachers dimensions primarily capture interpersonal and relational aspects of school alienation. This distinction is especially relevant in the Chinese university context, where students’ academic experiences are strongly organized around coursework, assessment, and self-regulated study, making alienation from learning a more direct indicator of academic disengagement. The subscale comprises eight items capturing students’ emotional disengagement, diminished interest, and perceived lack of meaning in academic learning (e.g., “I feel little enjoyment or satisfaction when learning at school”).

All substantive items were rated on five-point Likert scales ranging from 1 (strongly disagree) to 5 (strongly agree), with higher scores indicating higher levels of the respective constructs.

### 2.3 Data analysis

Statistical analyses were conducted using IBM SPSS and AMOS (Version 26). Descriptive statistics were first computed to examine the mean levels and variability of the measured constructs, thereby providing information about the distribution of scores and the extent of sample homogeneity or heterogeneity. Normality was also assessed using skewness and kurtosis values. The skewness values of the observed items ranged from −0.604 to 0.339, and the kurtosis values ranged from −0.907 to 0.341, indicating no substantial departure from univariate normality. Confirmatory factor analysis (CFA) was applied to evaluate the measurement model, including internal consistency and construct validity. Reliability was assessed using Cronbach’s alpha and composite reliability (CR), while structural equation modeling (SEM) tested the hypothesized relationships among mindfulness, cognitive fusion, psychological resilience, and academic alienation. Indirect effects were examined through bootstrapping with 5,000 resamples and 95% bias-corrected confidence intervals. Model fit was determined based on recommended fit indices.

To examine potential common method variance (CMV), a CFA-based single-factor test was conducted. Specifically, a single-factor measurement model was compared with the proposed multi-factor measurement model. The single-factor model showed poor fit to the data (*χ*^2^ = 12,177.583, df = 324, *p* < 0.001), whereas the proposed multi-factor model demonstrated a substantially better fit (*χ*^2^ = 1,050.291, df = 293, *p* < 0.001). This result indicates that the covariance among the study variables was not adequately explained by a single latent factor, suggesting that CMV was unlikely to pose a serious threat to the validity of the findings.

## 3 Results

### 3.1 Descriptive statistics and correlations

Descriptive statistics and correlations among the study variables are presented in Table 2. Overall, participants reported moderate levels of mindfulness (*M* = 3.594, SD = 0.745), cognitive fusion (*M* = 3.075, SD = 0.959), psychological resilience (*M* = 3.332, SD = 0.821), and academic alienation (*M* = 2.889, SD = 0.927). The standard deviation values indicate sufficient variability across the measured constructs, suggesting that the sample was not overly homogeneous and was appropriate for subsequent structural analyses. In addition, mindfulness was positively associated with psychological resilience and negatively associated with cognitive fusion and academic alienation, whereas cognitive fusion was positively associated with academic alienation and negatively associated with psychological resilience. Psychological resilience was negatively associated with academic alienation.

**Table 2:** Descriptive statistics, correlations, and discriminant validity.

Construct	Mean	SD	1	2	3	4
Mindfulness (1)	3.594	0.745	(0.801)			
Cognitive Fusion (2)	3.075	0.959	−0.408**	(0.834)		
Psychological Resilience (3)	3.332	0.821	0.586**	−0.522**	(0.839)	
Academic Alienation (4)	2.889	0.927	−0.373**	0.500**	−0.426**	(0.831)

The square roots of the AVE are shown on the diagonal; the off-diagonal elements are Pearson correlations among the constructs. **p < 0.01.

### 3.2 Measurement model

As shown in Table 3, CFA results supported the adequacy of the measurement model. All standardized factor loadings exceeded 0.70, indicating satisfactory indicator reliability. Internal consistency was confirmed, with Cronbach’s alpha and composite reliability values above the recommended threshold of 0.70.

**Table 3:** Reliability and validity.

Items	Factor loadings	Cronbach’s α	CR	AVE
Mindfulness (MI)		0.899	0.899	0.641
MI1	0.769			
MI2	0.796			
MI3	0.820			
MI4	0.835			
MI5	0.781			
Cognitive Fusion (CF)		0.941	0.941	0.695
CF1	0.821			
CF2	0.818			
CF3	0.815			
CF4	0.840			
CF5	0.838			
CF6	0.868			
CF7	0.834			
Psychological Resilience (PR)		0.934	0.934	0.704
PR1	0.826			
PR2	0.830			
PR3	0.803			
PR4	0.868			
PR5	0.831			
PR6	0.874			
Academic Alienation (AA)		0.947	0.947	0.690
AA1	0.833			
AA2	0.888			
AA3	0.904			
AA4	0.901			
AA5	0.848			
AA6	0.773			
AA7	0.756			
AA8	0.722			

Convergent validity was evidenced by average variance extracted (AVE) values ranging from 0.641 to 0.704, exceeding the suggested cutoff of 0.50. Discriminant validity was established using the Fornell–Larcker criterion, as the square roots of the AVE values shown in Table 2 were greater than the corresponding inter-construct correlations. Overall, the measurement model demonstrated acceptable reliability and validity, supporting subsequent structural analyses.

### 3.3 Structural mod**el**

The structural model was examined using structural equation modeling (SEM) to test the hypothesized relationships among mindfulness, cognitive fusion, psychological resilience, and academic alienation. The overall model demonstrated an acceptable to good fit to the data, with *χ*^2^/df = 2.46, GFI = 0.911, AGFI = 0.893, and RMR = 0.046. Although the AGFI value (0.893) was slightly below the conventional 0.90 criterion, it was very close to this threshold and remained within the range commonly regarded as acceptable. In addition, because AGFI may be influenced by sample size and model complexity, model fit was evaluated based on the overall pattern of fit indices rather than any single indicator. In addition, the model showed a low RMSEA value (RMSEA = 0.051, 90% CI [0.046, 0.055], PCLOSE = 0.401), further supporting the adequacy of model fit. Incremental fit indices also exceeded recommended thresholds (CFI = 0.966, TLI = 0.962, IFI = 0.966, and NFI = 0.944), indicating that the proposed structural model adequately represented the observed data.

**Figure 2:**
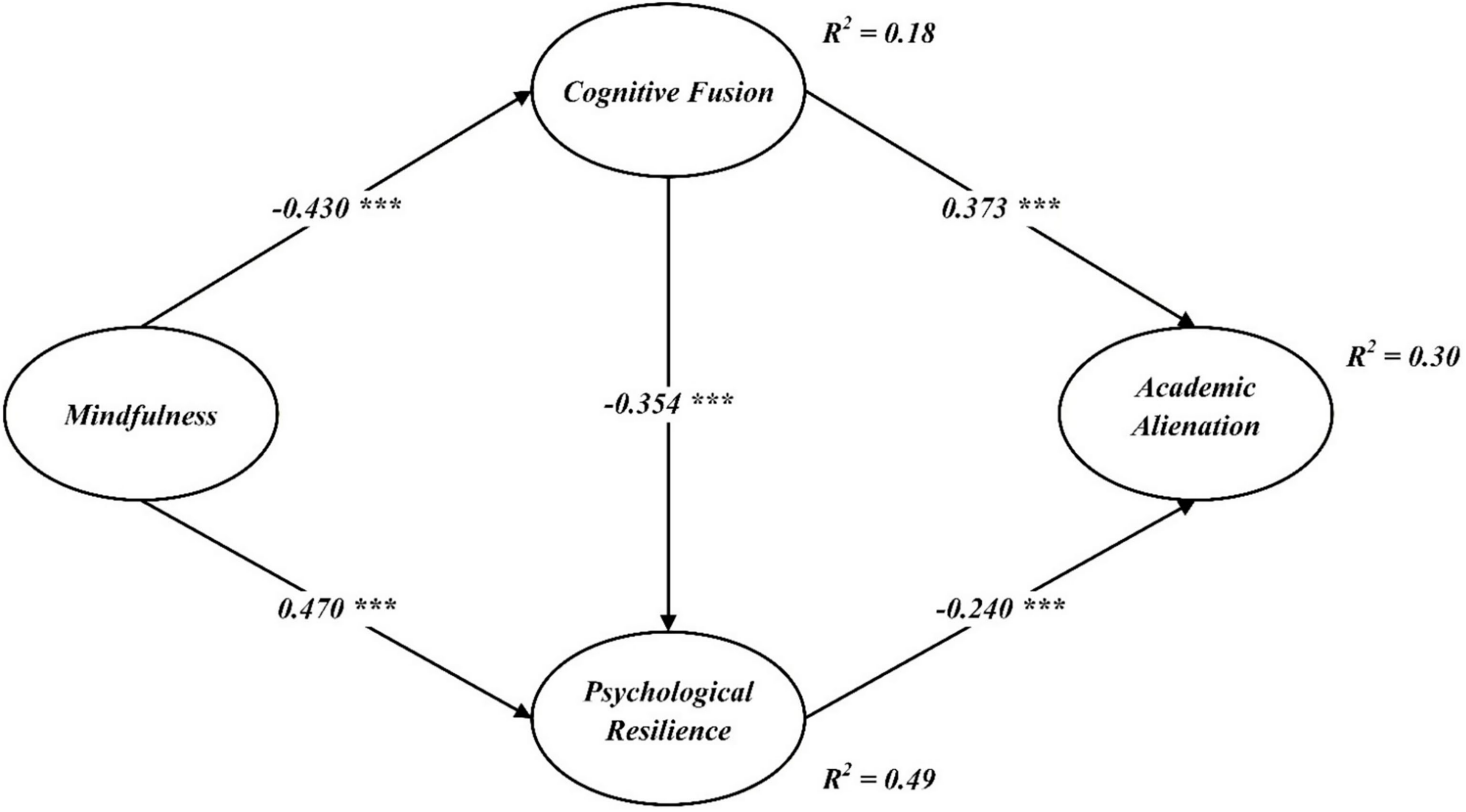
Structural path model. *** *p* < 0.001.

As shown in Figure 2, all hypothesized direct paths were statistically significant and in the expected directions. Specifically, mindfulness was negatively associated with cognitive fusion (*β* = −0.430, *p* < 0.001) and positively associated with psychological resilience (*β* = 0.470, *p* < 0.001), supporting H1 and H2, respectively. Moreover, cognitive fusion was negatively related to psychological resilience (*β* = −0.354, *p* < 0.001), supporting H3. With respect to academic alienation, cognitive fusion showed a positive association (*β* = 0.373, *p* < 0.001), whereas psychological resilience was negatively associated with academic alienation (*β* = −0.240, *p* < 0.001), providing support for H4 and H5.

The model accounted for 18% of the variance in cognitive fusion, 49% of the variance in psychological resilience, and 30% of the variance in academic alienation, indicating satisfactory explanatory power.

To further test H6, a bootstrapping procedure with 5,000 resamples was employed to examine the mediating effects of cognitive fusion and psychological resilience. As reported in Table 4, the total indirect effect of mindfulness on academic alienation was statistically significant [*β* = −0.310, SE = 0.035, *Z* = −8.857, 95% bias-corrected CI (−0.383, −0.244), *p* < 0.001], with the confidence interval excluding zero. These results indicate that cognitive fusion and psychological resilience mediate the relationship between mindfulness and academic alienation. Specifically, higher mindfulness was associated with lower cognitive fusion, which in turn predicted greater psychological resilience and, ultimately, reduced academic alienation.

**Table 4:** Standardized indirect effect.

Path	Point estimate	Product of coefficients		Bootstrapping		
				Bias-corrected 95% CI		Two-tailed significance
		SE	*Z*	Lower	Upper	
MI → AA	−0.310	0.035	−8.857	−0.383	−0.244	*p* < 0.001

## 4 Discussion

### 4.1 Theoretical contributions

This study makes an important supplement to the theoretical analysis of academic alienation among university students. First, previous studies on academic alienation have mainly focused on social factors (Atila, 2023; Ghalavandi, 2023; Hadjar and Backes, 2023; Poutanen, 2023; Atiq et al., 2025) and psychological factors (Chang et al., 2025; Adam, 2023), but few studies have systematically explored the psychological mechanism between mindfulness and academic alienation from the perspective of mindfulness. This study integrates a structural model of mindfulness, cognitive fusion, and psychological resilience, and introduces a mindfulness-based cognitive regulation pathway on the basis of existing theoretical frameworks, thereby expanding the explanatory perspective of academic alienation.

The results of this study support the view that psychological resilience has a significant impact on academic alienation among university students (Khangura et al., 2020a), and further verify that cognitive fusion is an important psychological mechanism affecting academic alienation. This study emphasizes that, under the background of long-term academic pressure, university students are more likely to experience a state of cognitive fusion (Miniati et al., 2023). This state may lead to cognitive rigidity and emotional distress, making it difficult for individuals to effectively carry out emotional regulation and cognitive reappraisal when facing setbacks. When individuals are unable to respond flexibly to difficulties, frustration and powerlessness may accumulate continuously, thereby aggravating anxiety and psychological stress, and further strengthening the tendency toward academic alienation.

Second, the present study clarifies the structural links among mindfulness, cognitive fusion, psychological resilience, and academic alienation in university students. The results indicate that mindfulness is inversely associated with cognitive fusion and positively associated with psychological resilience, aligning with prior empirical evidence (Tanay et al., 2012; Zhang et al., 2023; Pidgeon and Keye, 2014). Among the examined variables, mindfulness exerted the strongest influence on resilience, followed by cognitive fusion. Both cognitive fusion and psychological resilience functioned as mediators in the association between mindfulness and academic alienation. Collectively, the proposed model accounted for 30% of the variance in academic alienation, suggesting meaningful explanatory capacity.

From a broader perspective, these findings also offer theoretical implications for the intersection of cognitive science and higher education research. By conceptualizing academic alienation as a consequence of regulatory imbalance in cognitive and emotional processing, the study contributes to emerging efforts to integrate cognitive regulation frameworks into educational well-being research. Moreover, the results provide individual-level evidence relevant to SDG 3 (Good Health and Well-Being) and SDG 4 (Quality Education), suggesting that enhancing mindfulness and resilience may represent theoretically grounded avenues for promoting sustainable academic engagement.

### 4.2 Practical implications

Considering the influence of mindfulness on cognitive fusion and psychological resilience, as well as its indirect effect on reducing academic alienation among university students, this study proposes the following practical implications.

At the societal level, it is recommended that mental health service institutions develop digital tools based on mindfulness training. Considering university students’ limited time and financial resources, as well as their high dependence on mobile devices, mobile-based mindfulness interventions (such as mindfulness meditation apps) may become a low-cost and sustainable form of psychological support (Flett et al., 2019). Mental health service institutions can combine online courses with counseling services to build an intervention system integrating digital and offline support, thereby expanding service coverage and improving the accessibility and sustainability of interventions.

At the school level, first, it is recommended that universities incorporate mindfulness training into the curriculum system. Research shows that students who integrate mindfulness into their courses may gain benefits in both academic and personal development. Through mindfulness training, university students can improve their level of mindfulness, enabling them to be more clear-headed and aware, and to focus on present tasks and goals rather than being disturbed by emotions (de Bruin et al., 2015). The content of mindfulness courses may include mindfulness meditation, breathing exercises, emotional management skills, and stress coping strategies, helping students establish healthy psychological regulation mechanisms. Such courses can be arranged in mental health education classes for first-year students, so that students can master stress-coping skills from the very beginning. Second, mindfulness training can be integrated into daily teaching and activities (Siegel, 2010). Before the start of each class, teachers can guide students in brief mindfulness meditation or breathing exercises to help them relax and improve concentration. Schools can also organize mindfulness meditation groups or related lectures in extracurricular activities, regularly providing students with opportunities for meditation and relaxation, and cultivating students’ habits of self-regulation.

At the individual level, mindfulness practices may be incorporated into everyday routines, such as mindful breathing or cultivating awareness of present-moment experiences, to enhance tolerance of stress and setbacks. Such practices can attenuate emotional reactivity and support greater psychological stability when confronting academic challenges (Siegel, 2010). In addition, structured self-reflection strategies may be beneficial. For example, resilience-focused journaling involves documenting daily stressors, emotional responses, coping strategies, and outcomes, thereby facilitating improved emotional regulation and stress management over time (Lohner and Aprea, 2021). Sustained engagement in these practices may gradually strengthen psychological resilience. Timely support is also important. When academic alienation emerges or begins to undermine learning engagement and mental health, students are encouraged to communicate with academic advisors or counseling services. Accessing professional psychological support, when appropriate, may help students better understand their emotional and behavioral patterns and develop more adaptive coping strategies.

### 4.3 Limitations

Several limitations should be acknowledged. First, the proposed model did not incorporate potential moderating factors, such as personality traits, emotional intelligence, or self-efficacy, which could influence the associations among mindfulness, cognitive fusion, psychological resilience, and academic alienation. Future investigations may consider incorporating moderated mediation frameworks to provide a more comprehensive theoretical explanation.

Second, the cross-sectional design and the use of convenience sampling limit both the causal interpretation and the generalizability of the findings. Because the data were collected from universities within Hunan Province using a non-probability sampling approach, conclusions regarding temporal ordering remain tentative and the external validity of the results may be constrained. Future studies are encouraged to adopt longitudinal or experimental designs and to recruit more diverse samples from broader regions to strengthen both internal and external validity.

Third, although basic inclusion and exclusion criteria were applied, the sample was not screened with regard to participants’ mental health status. As a result, students with substantial psychological distress or other mental health-related outlying characteristics may have been included in the sample, which may have introduced unmeasured heterogeneity into the findings. Future studies are encouraged to incorporate more refined screening procedures or mental health-related control measures in order to improve sample characterization and the interpretation of results.

## 5 Conclusion

Based on the research objectives and empirical results, this study suggests that mindfulness, cognitive fusion, and psychological resilience are important factors associated with academic alienation among university students. Specifically, mindfulness may be indirectly associated with lower academic alienation through lower levels of cognitive fusion and higher psychological resilience. This pathway highlights the potential relevance of mindfulness in cognitive regulation and psychological adaptation. Therefore, universities may consider systematically promoting mindfulness-related courses and psychological support programmes, while encouraging students to participate in mindfulness training to enhance self-awareness and stress-coping abilities. For students who already exhibit marked academic alienation, timely professional support may help promote their mental health and academic development.

## Data Availability

The raw data supporting the conclusions of this article will be made available by the authors, without undue reservation.
